# Gravure‐Printed Flexible Perovskite Solar Cells: Toward Roll‐to‐Roll Manufacturing

**DOI:** 10.1002/advs.201802094

**Published:** 2019-01-28

**Authors:** Young Yun Kim, Tae‐Youl Yang, Riikka Suhonen, Marja Välimäki, Tiina Maaninen, Antti Kemppainen, Nam Joong Jeon, Jangwon Seo

**Affiliations:** ^1^ Division of Advanced Materials Korea Research Institute of Chemical Technology (KRICT) 141 Gajeong‐ro Yuseong‐gu Daejeon 34114 Republic of Korea; ^2^ Printed Electronics Processing VTT Technical Research Centre of Finland Ltd. Kaitoväylä 1 Oulu 90571 Finland

**Keywords:** flexible perovskite solar cells, gravure printing, perovskite solar cells, roll‐to‐roll process

## Abstract

Recent advances in perovskite solar cells (PSCs) have resulted in greater than 23% efficiency with superior advantages such as flexibility and solution‐processability, allowing PSCs to be fabricated by a high‐throughput and low‐cost roll‐to‐roll (R2R) process. The development of scalable deposition processes is crucial to realize R2R production of flexible PSCs. Gravure printing is a promising candidate with the benefit of direct printing of the desired layer with arbitrary shape and size by using the R2R process. Here, flexible PSCs are fabricated by gravure printing. Printing inks and processing parameters are optimized to obtain smooth and uniform films. SnO_2_ nanoparticles are uniformly printed by reducing surface tension. Perovskite layers are successfully formed by optimizing the printing parameters and subsequent antisolvent bathing. 2,2′,7,7′‐Tetrakis‐(*N*,*N*‐di‐4‐methoxyphenylamino)‐9,9′‐spirobifluorene is also successfully printed. The all‐gravure‐printed device exhibits 17.2% champion efficiency, with 15.5% maximum power point tracking efficiency for 1000 s. Gravure‐printed flexible PSCs based on a two‐step deposition of perovskite layer are also demonstrated. Furthermore, a R2R process based on the gravure printing is demonstrated. The champion efficiency of 9.7% is achieved for partly R2R‐processed PSCs based on a two‐step fabrication of the perovskite layer.

## 1 Introduction

The technologies of perovskite solar cells (PSCs) have matured to the point where they can be commercialized at a laboratory scale.^1^, ^2^, ^3^, ^4^, ^5^, ^6^, ^7^ The power conversion efficiency (PCE) of PSCs has rapidly increased up to 23%, which is comparable with that of commercialized inorganic thin film photovoltaics such as CdTe and Cu(In,Ga)(S,Se) solar cells.^8^, ^9^, ^10^ Meanwhile, remarkable progress in the long‐term operational stability of PSCs has been made by introducing durable perovskite composition, interlayers, and encapsulation.^4^, ^11^, ^12^, ^13^, ^14^, ^15^, ^16^, ^17^, ^18^, ^19^, ^20^, ^21^, ^22^, ^23^ In addition, PSCs possess unique advantages such as solution‐processability and flexibility compared to the inorganic thin film photovoltaics.^24^, ^25^, ^26^

In order to transfer these technologies from laboratory to industrial scale, large‐area PSC manufacturing using scalable coating or printing processes has been developed; slot‐die coating,^27^, ^28^ blade coating,^29^, ^30^, ^31^, ^32^ screen printing,^33^ and ink‐jet printing^34^, ^35^ of the perovskite layer have been successfully demonstrated. Coating methods are commonly used to form a layer over an entire area or simple 1D stripe patterns on substrates. Printing methods are applied for direct coating and patterning on substrates at once, thereby providing a large degree of freedom for module design with arbitrary shapes and sizes.^36^, ^37^, ^38^ In particular, gravure printing offers an efficient way to directly print the desired patterned layer with high throughput at fast printing speed.^38^, ^39^, ^40^, ^41^, ^42^, ^43^, ^44^ Notwithstanding these advantages, there still have been no attempts to make PSCs based on gravure printing despite that gravure printing of custom‐shaped organic photovoltaics (OPVs) was demonstrated previously.^38^, ^42^, ^43^

Additionally, gravure printing is a roll‐to‐roll (R2R) compatible deposition method enabling cost‐effective and high‐throughput industrial‐scale production of PSCs on flexible substrates. Recently, the R2R fabrication of PSCs has been reported in several literatures.^45^, ^46^, ^47^, ^48^ In addition, Solliance announced the demonstration of R2R process for PSCs.^49^ The PCEs of the resulting devices were from 11.2 to 14.1% from a small area (<0.1 cm^2^), respectively. However, there is still room for improvement for R2R‐processed PSCs in terms of PCE and stability.

To fully demonstrate efficient PSCs by scalable methods and a R2R process, it is essential to deposit a uniform and pinhole‐free perovskite film as well as selective charge transporting layers by low‐temperature processes. There are two methods for fabrication of perovskite film with high quality. One‐step method, also known as the solvent engineering method utilizes mediator molecules to retard crystallization of perovskite precursors. A uniform layer is formed by antisolvent dripping or bathing.^3^, ^50^ In the two‐step method, a PbI_2_ film is deposited first, and this is converted to perovskite by contacting with organic ammonium “halide” molecules in solution.^18^, ^51^ For the one‐step method, many studies reported that smooth and uniform films can be produced in a small area with antisolvent dripping at a specific time during process. For large‐area application, especially with the R2R process, antisolvent bathing should be finely optimized to achieve PSCs with a high PCE. The processing window for the two‐step method is wider and for that reason it is easier to scale up the deposition process for this method even though it requires more processing steps and thus more processing time. The processing time of the two‐step method was shortened for high‐throughput fabrication by introducing mediator extraction treatment (MET) in our previous study.^51^ Moreover, the processing solvents (such as 2‐propanol (IPA) or mixture of IPA) are less harmful to the human health.

In order to deposit charge transporting layers by scalable methods and furthermore by a R2R process on a flexible substrate, a p‐i‐n structure with organic transport materials has generally been employed.^29^, ^42^, ^45^ This is mainly because all the layers in the p‐i‐n structure are already well‐designed for scalable fabrication methods from OPVs.^52^ However, in terms of PCE and long‐term stability, the PSCs using a n‐i‐p structure exhibit better performance due to the use of metal oxide electron transport layers (ETLs). The only hurdle to be crossed is the processing temperature. Metal oxide electron transport layers such as TiO_2_ are typically annealed at temperatures as high as 500 °C. As an alternative, SnO_2_ is one of the representative ETL materials for n‐i‐p structure, and it can be formed at relatively lower temperature by a sol‐gel method or chemical bath deposition.^53^, ^54^ In particular, SnO_2_ nanoparticles recently have been highlighted as an ETL because they can be deposited at a temperature as low as 100 °C. This temperature is applicable to continuous R2R processes based on flexible substrates, and a device with SnO_2_ nanoparticles displayed a high PCE.^55^, ^56^ However, because SnO_2_ should be much thinner than TiO_2_ to achieve a high open‐circuit voltage (*V*_oc_) and small hysteresis, uniform deposition of SnO_2_ on a large‐area substrate is more challenging.

In this work, we demonstrate for the first time the fabrication of all‐printed PSCs with a n‐i‐p structure on flexible substrates using gravure printing. In order to deposit uniform films by gravure printing, the properties of each solution and the processing conditions for the fabrication under ambient conditions were optimized. A thin layer of SnO_2_ nanoparticles as an ETL was uniformly printed on indium tin oxide (ITO) coated poly(ethylene terephthalate) (PET) substrates by adjusting the surface tension of the aqueous dispersion of SnO_2_ nanoparticles by mixing it with IPA. Methylammonium lead iodide (MAPbI_3_) films with excellent surface morphology were formed by using the one‐step method with optimized processing parameters, especially drying time before the antisolvent (diethyl ether) bathing. 2,2′,7,7′‐Tetrakis‐(*N*,*N*‐di‐4‐methoxyphenylamino)‐9,9′‐spirobifluorene (Spiro‐OMeTAD) was also successfully printed on top of the perovskite layer. Surprisingly, the resulting all‐printed devices showed a PCE of up to 17.2 %, which is the best PCE achieved for all‐printed PSCs. In addition, the fabrication of R2R‐printed PSCs was demonstrated by using gravure printing. Although the two‐step MET method was employed for the deposition of perovskite films, a high PCE of 9.7% was achieved by all‐printed, partly R2R‐based PSCs.

## 2 Result and Discussion

### 2.1 Gravure Printing of Perovskite Layer

**Figure 1:**
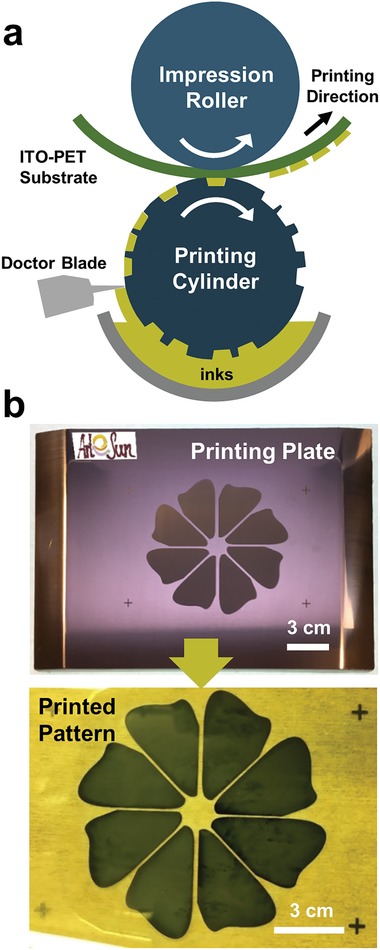
a) Schematic diagram represents gravure printing on the flexible substrate. b) The images of printing plate with flower‐shape engraved pattern and resulting printed pattern of perovskite on the PET substrate.

In the gravure printing, the ink is first applied to an engraved printing cylinder and then it is transferred to a substrate from the cylinder (**Figure** **1**a). During the printing process, the printing cylinder is partially immersed in the ink container and the engravings are filled with the ink. The cylinder has an engraved pattern that defines the active area for the ink transfer. A doctor blade is used to wipe the rotating cylinder to remove the excess ink from the inactive area of the printing cylinder. The ink is then transferred from the cylinder to a desired substrate by applying pressure with the impression roller. For the first time, all of the layers of perovskite solar cells except electrodes were deposited by successive gravure printing in this work. SnO_2_ nanoparticles were printed as the ETL, MAPbI_3_ was printed as the light‐absorbing layer, and Spiro‐OMeTAD and poly(3‐hexylthiophene) (P3HT) were printed as the hole transport layers (HTL).

**Figure 2:**
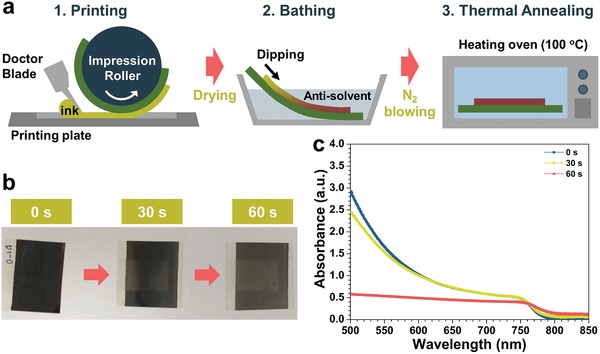
a) A schematic illustration of printing perovskite on the flexible substrate. b) Optical images of printed perovskite layer with different drying time before bathing. c) UV–vis absorption spectra of perovskite layer with different drying time.

Unlike various coating methods such as bar, blade, or slot‐die coating, the gravure printing technique can deposit and pattern a desired layer at once with high precision and resolution in a short time, even for complex and 2D patterns. In order to demonstrate this unique advantage of gravure printing, a perovskite layer with a customized flower pattern was printed as described in Figure 1b. A small amount of polymer, poly(vinylpyrrolidone) (PVP) was added to the precursor solution to further improve the resolution of the transferred pattern by increasing the viscosity of the solution. As shown in Figure 1b, the flower pattern composed of perovskite was successfully formed by gravure printing and subsequent bathing in antisolvent. This enabled the formation of a large‐area (over several tens of cm^2^), uniform perovskite layer by simple gravure printing of a precursor solution with a very small amount of the additive polymer for adjusting viscosity. The precise replication of pattern with a gap of 1–2 mm is also possible by gravure printing. In this case, a sheet‐based, table‐top gravure printer was used. This printer is operated with a patterned printing plate instead of a printing cylinder, as displayed in **Figure** **2**a. First, the perovskite precursor solution was dispensed directly on the printing plate, and then the solution was wiped by a doctor blade to fill the active areas of the printing plate. A perovskite intermediate film was formed on the flexible substrate by transfer of the precursor ink from printing plate and pressing with an impression roller. By this simple gravure printing process and subsequent crystallization process, a well‐defined flower pattern of MAPbI_3_ on a flexible substrate without any additional patterning steps was formed thereby demonstrating excellent patterning properties of the perovskite layer in the gravure printing process.

The crystallization process was newly designed to form a high‐quality perovskite layer after the printing. For the fabrication of OPVs, a polymer light‐absorbing layer can be formed by simple drying of the printed wet film. However, the perovskite layer requires not only a drying process, but also a phase transition of the intermediate phase to the perovskite phase with controlled crystallization behavior. The well‐established one‐step method for spin‐coating includes the dripping of an antisolvent during the spin‐coating of the precursor solution containing the mediator solvent, dimethyl sulfoxide (DMSO). Considering a large‐area fabrication and a R2R process, the dripping of an antisolvent must be changed to an antisolvent bathing. It is noteworthy that both the formation of the wet thin layer and supersaturation by evaporation of the processing solvent take place simultaneously during the spin‐coating, while these happen independently in the printing process. In addition, the dripping includes a physical stream of the antisolvent, but the bathing does not. A detailed procedure for printing and formation of perovskite layer is illustrated in Figure 2a. The precursor‐printed substrate is dried and immersed into the antisolvent bath to remove the excess solvent and the mediator molecules, DMSO. The film is then placed in the heating oven at 100 °C for 10 min after being blown by N_2_.

The time required for each procedure and the time between each procedure were controlled to achieve an optimized perovskite film. The most critical timescale was the drying time before the antisolvent bathing. It is noted that =20 s was required to collect the printed substrate from the printing plate. After collecting the substrate, the printed film began to dry completely and to change to a white color starting from the edges of the printed patterns. As the drying time was prolonged, the perovskite intermediate film was transformed to a white and hazy appearance. Even after the antisolvent bathing, the films formed with a prolonged drying time appeared hazy and light gray in color rather than black, as shown in Figure 2b. The best quality of the perovskite films could be obtained when the collected substrates were immersed into the diethyl ether bath as quickly as possible (0 s). This difference is also revealed in the UV–vis absorption spectra (Figure 2c). The film formed by immediate immersion in the antisolvent bath after collecting the substrate showed the characteristic absorption spectra of MAPbI_3_ with an onset at around 800 nm. The perovskite formed after an additional drying time of 30 s showed slightly decreased absorption with increased roughness revealed at an increased absorbance above 800 nm. The drying time of 60 s resulted in the formation of a film with the most reduced absorbance, which means that the perovskite cannot act as a good light absorber. The morphology of the resulting film appeared very rough, as an entangled network of wires (Figure S1, Supporting Information). The duration of bathing and the time interval between the bathing and the thermal annealing were also varied, but the variation of these process parameters did not affect the film quality of the perovskite.

**Figure 3:**
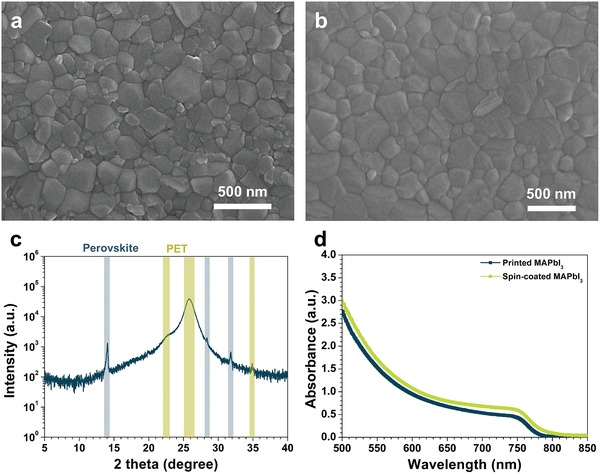
a,b) SEM images of perovskite layer formed by spin‐coating and gravure printing. c) XRD spectrum of perovskite layer printed on PET substrate. d) UV–vis spectra of perovskite formed by spin‐coating and gravure printing.

The perovskite layers formed by spin‐coating and gravure printing have a similar morphology, as observed by a scanning electron microscope (SEM) (**Figure** **3**a,b). A pinhole‐free, densely packed perovskite film was obtained by gravure printing. The root‐mean‐square roughness was calculated as 19.4 nm, from atomic force microscope (AFM) image (Figure S2b, Supporting Information). Previous works claimed that the film deposited by gravure printing shows random voided areas with a thickness variation resembling the engraved pattern of the printing plate.^57^ However, the gravure‐printed perovskite film in this work shows a very uniform, dense, and pinhole‐free film morphology across the entire surface, as confirmed by low‐magnification SEM images (Figure S3, Supporting Information).

The X‐ray diffraction (XRD) spectrum of the printed perovskite film showed distinct peaks at 14° and 28°, indicating the (101) and (202) planes of MAPbI_3_ (Figure 3c). In addition, there are no observable peaks from PbI_2_, which is an indication of complete conversion of the printed precursors to the MAPbI_3_ perovskite. The printed and the spin‐coated MAPbI_3_ have almost the same absorption spectra (Figure 3d). This finding indicates that both the thickness and the quality of the MAPbI_3_ layer formed by gravure printing are quite similar to those formed by spin‐coating.

### 2.2 Fabrication of Flexible PSCs by Gravure Printing with the One‐Step Method

**Figure 4:**
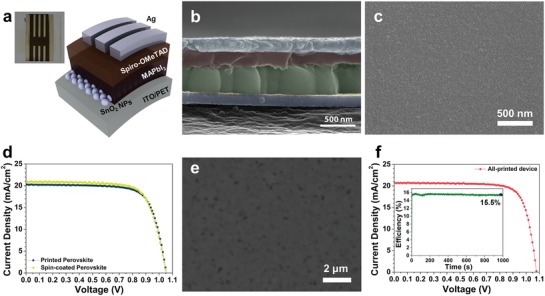
a) Schematic diagram of the structure of flexible solar cells made by gravure printing. (Inset) A picture of flexible solar cell. b) Cross‐sectional SEM image of flexible perovskite solar cell. Each layers are colored with different color. (Blue: ITO, yellow: SnO_2_, green: perovskite, red: Spiro‐OMeTAD, light blue: Ag.) c) SEM image of SnO_2_ layer deposited on the ITO/PET. d) *J*–*V* curves of flexible perovskite solar cells. e) SEM image of printed Spiro‐OMeTAD on the top of perovskite/SnO_2_/ITO/PET. f) *J*–*V* curve of all‐printed flexible perovskite solar cell. (Inset) MPPT curve for all‐printed device.

The flexible PSCs were fabricated by gravure printing. The device structure is illustrated in **Figure** **4**a. SnO_2_, MAPbI_3_, and Spiro‐OMeTAD were sequentially printed on the prepatterned ITO‐coated PET flexible substrate. Because all the layers can be processed at low temperature, the device can be deposited on a PET substrate. Only the Ag electrodes were formed by thermal evaporation as shown in the inset of Figure 4a. The actual device area is defined by covering the shadow mask on the device for measurement. By gravure printing, all the layers were well‐formed without any cross‐dissolution or void formation (Figure 4b). The thicknesses of the layers in the all‐printed device were measured in the cross‐sectional SEM image. The thicknesses of ITO, SnO_2_ nanoparticles, MAPbI_3_, Spiro‐OMeTAD, and Ag were 140, 20, 380, 220, and 200 nm, respectively.

For the printing of the SnO_2_ layer, a colloidal dispersion of SnO_2_ nanoparticles dispersed in water was used. High surface tension of water can result in poor uniformity of the printed films because of ribbing or coffee‐ring effects. In order to adjust the thickness of the SnO_2_ layer and reduce the surface tension of the solution, a mixed solvent of water and IPA was used. The printing of the diluted SnO_2_ nanoparticles followed by drying at 120 °C for 10 min produced a very uniform, conformal, and pinhole‐free film on the substrate (Figure 4c and Figure S2a, Supporting Information). The average surface roughness of the film was 1.7 nm.

The perovskite layer was then gravure printed on top of the printed SnO_2_ layer as described in Figure 2a. The performance of the device with a printed perovskite layer was confirmed by comparing it to the device with a spin‐coated perovskite layer. Spiro‐OMeTAD was spin‐coated as a HTL on both devices for this comparison. Figure 4d shows the current density–voltage (*J*–*V*) curves of the flexible PSCs where the perovskite layer is processed either by gravure printing or by spin‐coating. The photovoltaic performance of the device with the printed perovskite was on par with that of the device with a spin‐coated perovskite. The PCEs of the champion devices with a spin‐coated perovskite and a printed perovskite were 16.1 and 15.7%, respectively. The devices show only a minor deviation in the short‐circuit current (*J*_sc_), whereas the open‐circuit voltage (*V*_oc_) and the fill factor are almost at the same level (**Table** **1**). As confirmed previously, prolonged drying of the printed perovskite precursor film resulted in poor photovoltaic performance, and changes in the dipping time or the time between dipping and annealing did not affect the performance of the resulting device (Figure S4 and Table S1, Supporting Information). In previous work, the perovskite layer formed by scalable processes other than gravure printing typically requires mixing surfactants in the solution or heating the substrate to achieve high uniformity and quality of the perovskite layer.^31^, ^32^ By using gravure printing, however, a uniform and high‐quality perovskite layer was successfully formed, and comparable photovoltaic performance was achieved without any additives in the precursor solution or elevating the temperature of the substrate.

**Table 1:** The solar parameters of the flexible PSCs

Samples	*V*_oc_ a) [V]	*J*_sc_ a) [mA cm^−2^]	Fill factora) [%]	Efficiencya) [%]
One‐step processed perovskite solar cells				
Spin‐coated perovskite/spin‐coated Spiro‐b)	1.05 (1.05 ± 0.03)	21.0 (20.7 ± 0.5)	72.3 (68.9 ± 2.83)	16.1 (14.9 ± 0.8)
Printed perovskite/spin‐coated Spiro‐b)	1.05 (1.04 ± 0.02)	20.3 (20.1 ± 0.3)	73.1 (70.4 ± 2.68)	15.7 (14.7 ± 0.7)
All‐printed (printed perovskite/printed Spiro‐)b)	1.07 (1.05 ± 0.03)	20.7 (20.5 ± 0.5)	77.1 (74.4 ± 1.95)	17.2 (16.0 ± 0.7)
Printed perovskite/printed P3HT	0.92	19.1	62.7	11.0
Two‐step processed perovskite solar cells				
Printed perovskite/spin‐coated Spiro‐	1.05	20.1	68.8	14.6
Printed perovskite/printed Spiro‐	0.95	19.6	58.9	10.9
Roll‐to‐roll, two‐step processed perovskite solar cells				
R2R‐printed perovskite/printed P3HT	0.89	17.2	63.1	9.7
R2R‐printed perovskite/printed Spiro‐	0.83	15.5	60.7	7.8

^a)^ Structure: PET/ITO/printed SnO_2_/spin‐coated or printed perovskite/spin‐coated or printed HTL (Spiro‐OMeTAD or P3HT)/Ag

^b)^ Averaged parameters are presented in the parenthesis.

As the next step, Spiro‐OMeTAD as a HTL was also deposited by gravure printing on top of the perovskite layer. The printed Spiro‐OMeTAD films showed a smooth morphology with excellent coverage on the perovskite layer (Figure 4e and Figure S2c, Supporting Information). The root‐mean‐square average surface roughness of the printed Spiro‐OMeTAD was 2.1 nm, indicating full coverage of Spiro‐OMeTAD on the perovskite without any shunt pathways.

Unexpectedly, the all‐printed device showed the highest champion PCE of 17.2% from a reverse scan, with an improved *V*_oc_ of 1.07 V and a fill factor of 77.1% (Figure 4f and Table 1). To the best of our knowledge, this is the highest PCE obtained from all‐printed PSCs.^27^, ^58^, ^59^, ^60^ The average PCE of the devices with spin‐coated and gravure‐printed Spiro‐OMeTAD on top of the printed perovskite layer was 14.7 ± 0.7 and 16.0 ± 0.7%, respectively (Table 1 and Figure S5, Supporting Information, averaged for 20 devices each). Increases in the *V*_oc_ and the fill factor may imply that the enhanced PCE originates from a better interfacial contact between the perovskite and the Spiro‐OMeTAD, which might result from mechanical pressure by the impression roller during the gravure printing. The external quantum efficiency (EQE) spectrum was measured for the all‐printed device (Figure S6, Supporting Information). The integration of the EQE curve gives 20.5 mA cm^−2^ of *J*_sc_, which is consistent with the result obtained from the *J*–*V* curve. Maximum powerpoint tracking (MPPT) was performed for the all‐printed solar cell in order to obtain a more realistic efficiency of the device and to investigate the light‐soaking stability (inset of Figure 4f). The MPPT efficiency of the best all‐printed flexible solar cell was 15.5%. Notably, the MPPT efficiency was retained over 1000 s, showing an excellent light‐soaking stability during the measurement, considering that the device was fabricated on a flexible substrate in ambient air and the measurement was also conducted in ambient air (23 °C, ≈30% RH). After continuous illumination over 2 h, the MPPT efficiency begins to drop rapidly. By encapsulation of the device, however, the device can retain its MPPT efficiency with minor drop of only 4% after 10 h (Figure S7, Supporting Information). Therefore, the gravure printing of flexible solar cells showed not only excellent applicability to the formation of various types of layers constituting PSCs, but also superior efficiency and light‐soaking stability.

### 2.3 Demonstration of Gravure Printing with the Two‐Step MET Method

In addition to the gravure printing of the perovskite layer based on the one‐step method, a two‐step method was also applied in order to use a relatively green and healthy solvent rather than the toxic and flammable solvent commonly used as an antisolvent in the one‐step process. Considering the R2R process, which requires a large amount of solvents, it is desirable to limit the usage of toxic and flammable solvents if the laboratory environment is not fully protected from such hazards.

Previously, we developed a modified two‐step method, MET, to reduce the preparation time of the perovskite layer.^51^ In the MET, a PbI_2_‐DMSO complex is first deposited on the desired substrate. The DMSO mediator is then efficiently extracted by IPA to produce a porous PbI_2_ film. The perovskite film is completed by simple dipping of the porous PbI_2_ film into methylammonium iodide (MAI) dissolved in IPA. In this case, the only solvent used is IPA, which is not harmful to the human health.

**Figure 5:**
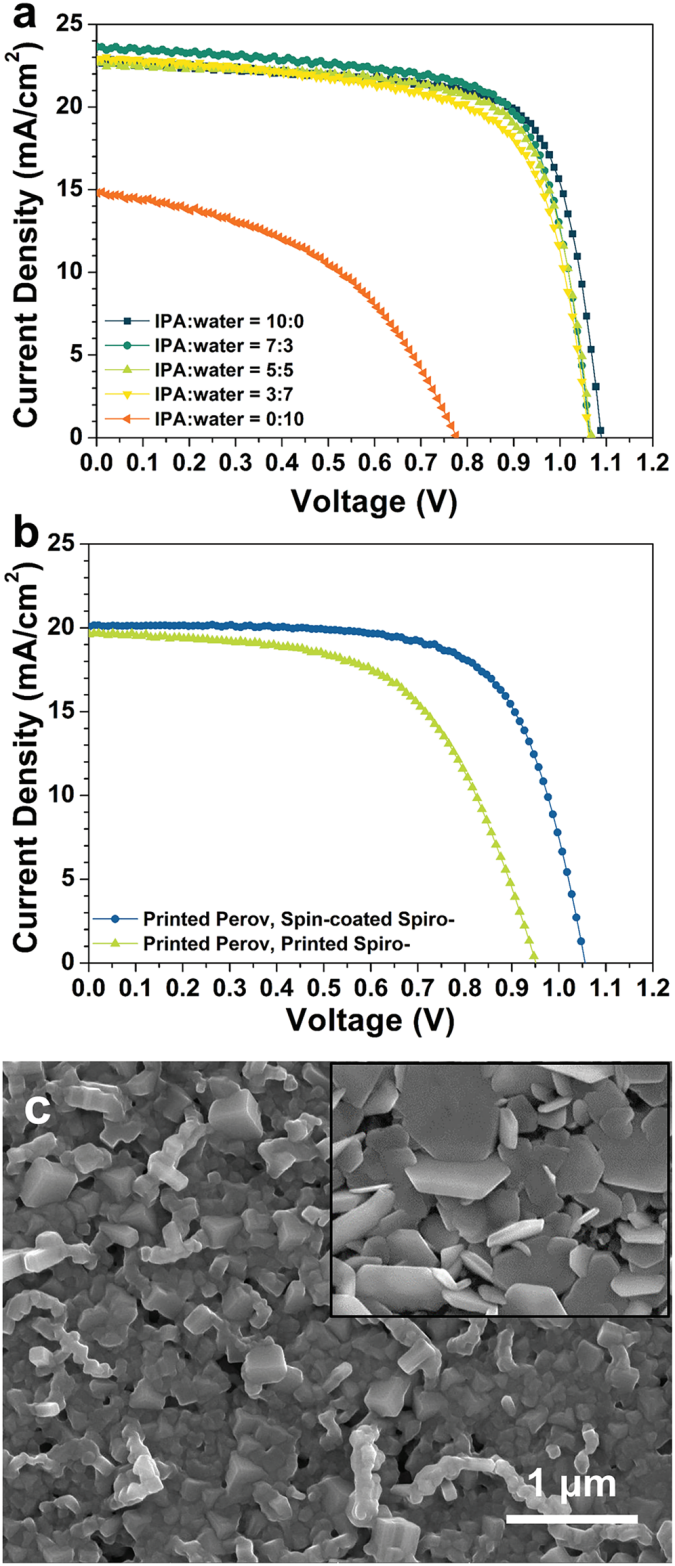
a) *J*–*V* curves of perovskite solar cells made by two‐step process with different ratio of mixed dipping solvent. The devices were fabricated on a glass/ITO substrate. b) *J*–*V* curves of printed perovskite solar cells fabricated by two‐step process. c) SEM image of printed perovskite by two‐step process. (Inset) SEM image of gravure‐printed PbI_2_.

Here, in order to reduce the flammability, we additionally mixed water with IPA in the DMSO‐extraction step. The mixing of water, a more polar solvent, into IPA resulted in the formation of more porous PbI_2_ films.^51^ Therefore, it is also expected that the mixing of solvent minimizes the time required for the extraction of the DMSO from the PbI_2_‐DMSO complex film and maximizes the reaction of PbI_2_ with MAI molecules within the given time. The effect of mixing water into IPA was investigated by comparing the performance of PSCs processed by using different ratios of IPA to water (**Figure** **5**a). The PSCs were fabricated on the ITO‐coated glass substrate. The results of the photovoltaic parameters are summarized in Table S2 (Supporting Information).

Although a slight decrease of the *V*_oc_ and the fill factor was observed with increasing the amount of water in the solvent mixture, the PCE of the devices showed reasonable values up to a 5:5 volume ratio of water to IPA. In addition, the extraction time of the DMSO mediator could be reduced, thus allowing an increased web speed in the R2R printing run by using the 5:5 mixture of water and IPA. Thus, we chose a 1:1 mixture of water and IPA as a extraction solvent, where the device exhibited a reasonable PCE without significantly sacrificing the performance for the fabrication of two‐step processed, gravure‐printed flexible PSCs.

Gravure‐printed, flexible PSCs based on the two‐step process were fabricated in a laboratory scale, before extending this technique into a large‐scale R2R process (Figure 5b and Table 1). The two‐step processed, gravure‐printed devices exhibited a PCE of 14.6%, which is comparable to that of one‐step processed, gravure‐printed solar cells. From this result, the two‐step process is verified as an effective method to fabricate the perovskite layer by gravure printing. We also attempted to print a Spiro‐OMeTAD layer on top of the perovskite layer processed with the two‐step method. However, the PCE of the device with the printed Spiro‐OMeTAD decreased to 10.9%, possibly due to the rough surface of the underlying two‐step processed perovskite layer. The printed PbI_2_ and the converted perovskite were observed through SEM (Figure 5c and inset). The PbI_2_ generated by bathing in IPA and water showed a flake‐like rough morphology with high porosity, and is all converted to perovskite rapidly with small voids. The surface coverage of the printed Spiro‐OMeTAD layer appears to be poorer than that of spin‐coated Spiro‐OMeTAD on the rough perovskite surface. Even though PCE of the devices with printed Spiro‐OMeTAD is much lower than that of the one‐step processed devices, we confirmed that the two‐step fabrication method is also an effective way for realizing a R2R printing process.

### 2.4 R2R Gravure Printing of Flexible PSCs

Based on the successful demonstration of gravure‐printed flexible PSCs using a table‐top printer in a laboratory, we applied the R2R gravure printing process to produce PSCs in a pilot scale. As mentioned earlier, the gravure‐printed device prepared by the two‐step process based on the modified MET showed lower photovoltaic performance in comparison to a device prepared by a one‐step process. Nevertheless, the two‐step fabrication process has some advantages with respect to continuous processing in a large scale in the ambient atmosphere. For example, this process utilizes environmentally safer processing solvents and has a wider processing window for the washing and curing of the printed layers.

**Figure 6:**
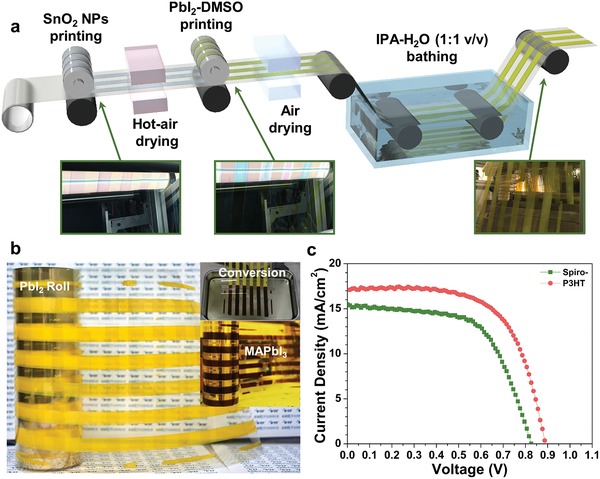
a) Schematic illustration of roll‐to‐roll process for perovskite solar cell. (Inset) Optical images of films wound on rolls. b) Image of PbI_2_ roll printed by roll‐to‐roll process. (Inset) Images of converting PbI_2_ to perovskite, and MAPbI_3_ roll. c) *J*–*V* curves of roll‐to‐roll printed solar cells with different HTLs.

The pilot‐scale R2R machine used in this work is presented in Figure S8 (Supporting Information). The whole procedure of R2R gravure printing is depicted in **Figure** **6**a. First, the SnO_2_ nanoparticles were R2R gravure printed on the top of the patterned PET/ITO film and then were dried by blowing hot air. The PbI_2_‐DMSO complex was subsequently R2R gravure printed on top of the SnO_2_‐coated PET/ITO substrate film. Next, the PbI_2_‐DMSO film was immersed in the bath containing a mixture of IPA and water (1:1 by volume) and is rapidly converted into a PbI_2_ film with yellow color upon removal of DMSO. The inset in Figure 6a presents images of the R2R‐printed substrate at each stage of the R2R gravure printing. Figure 6b displays a picture of the PbI_2_ roll prepared by the R2R printing process. The next step is immersion of the PbI_2_ film into a bath containing MAI solution with a high concentration and subsequent deposition of Spiro‐OMeTAD. Because of the high cost of the materials, the conversion and subsequent printing were carried out separately to be cost‐effective. To this end, the PbI_2_ layers were cut into sheets after R2R processing and dipped in the MAI bath to be converted to the perovskite (inset of Figure 6b). A Spiro‐OMeTAD or P3HT layer was gravure printed by a sheet‐based, lab‐scale gravure printer, followed by thermal evaporation of the Ag electrode to complete the device. The performance of the R2R processed solar cells is represented in Figure 6c and Table 1. When Spiro‐OMeTAD was used as the HTL, R2R processed solar cells showed a champion PCE of 7.8%, which is slightly decreased from the lab‐scale printed two‐step processed devices, which is 10.9% (Figure 5b and Table 1). This is likely due to the rougher morphology of the perovskite processed by two‐step method. Previous work suggested that Spiro‐OMeTAD films typically showed poor film morphology with pinholes and bad wettability due to the partial crystallization of itself and also by a large amount of additives.^61^, ^62^ The incomplete and defect‐rich film of Spiro‐OMeTAD on the rough, roll‐to‐roll processed perovskite layer maybe resulted in inefficient hole transport and electron blocking. Therefore, the roll‐to‐roll two‐step processed devices with printed Spiro‐OMeTAD showed reduced PCE with decreased *V*_oc_ by the increased amount of nonradiative recombination. In order to achieve better surface coverage on the perovskite layer, a uniform layer of HTL should be deposited on top of the perovskite layer. For this purpose, a polymer HTL, P3HT was gravure printed on top of the R2R processed perovskite layer. As a result, the solar cell with P3HT yielded a 9.7% champion PCE. For reference, one‐step processed PSCs with printed P3HT as HTL produced a 11.0% champion PCE (Figure S9, Supporting Information, and Table 1). The good quality of the R2R processed perovskite and the transporting layers is verified by the small deviation between the efficiency of our R2R processed device with printed P3HT, and that of a one‐step processed, lab‐scale printed device with P3HT. This result is especially encouraging and surprising because even all‐spin‐coated PSCs with P3HT HTL are typically reported to exhibit moderate PCEs from 10 to 17%.^63^, ^64^, ^65^ From this result, we verified the possibility of high‐throughput production of flexible PSCs by R2R gravure printing, with a reasonably high efficiency approaching almost 10%.

## 3 Conclusion

In this work, gravure printing was successfully applied for the fabrication of flexible PSCs for the first time. The printing inks and the processing parameters were optimized to achieve uniform deposition of the layers. For the ETL, a dispersion of SnO_2_ nanoparticles was diluted with a mixture of IPA and water, to reduce the surface tension and to obtain a smooth and pinhole‐free layer. MAPbI_3_ was formed by a printing step and subsequent antisolvent bathing. The effect of the timescale of each step and gaps between steps was investigated, and it was found that the drying time after the printing should be shortened to obtain a high‐quality perovskite layer processed by the one‐step method. The resulting perovskite layers showed very similar properties with the ones processed by spin‐coating, in terms of morphology, crystallography, and even photovoltaic performance. Spiro‐OMeTAD as a HTL was also successfully printed on top of the perovskite layer. As a result, all‐gravure‐printed flexible PSCs exhibited a champion PCE of 17.2%, which is superior to the PCE of the device with spin‐coated Spiro‐OMeTAD.

Based on the successful demonstration of the gravure printing at a lab‐scale, we also fabricated flexible PSCs by a continuous R2R process. For the experiment, the two‐step method allowing a wider operation window in terms of optimization of the processing parameters was utilized. As a result, a 9.7% PCE was achieved for the partially R2R processed devices. Based on a successful demonstration of gravure printing for the fabrication of PSCs at a lab‐scale and also by the R2R process, we believe this work suggests a new efficient and powerful method to fabricate PSCs. Gravure printing having unique advantages such as high throughput and ability to pattern with high resolution will provide a new possibility to extend the current success of PSCs into large‐area, commercial solar modules.

## 4 Experimental Section

*Formation of the Perovskite Layer*: A perovskite solution was prepared by dissolving 1.3 m of MAI (GreatcellSolar Ltd.) and PbI_2_ (TCI) in 0.8 mL of dimethylformamide (DMF) (Sigma‐Aldrich) and 0.1 mL of DMSO (Sigma‐Aldrich). The perovskite was formed by a multistep spin‐coating of the precursor solution at 500 rpm for 3 s, 1000 rpm for 3 s, and 5000 rpm for 50 s. 1 mL of diethyl ether was dripped during the final step of the spin‐coating. For gravure printing, 0.5 mL of the precursor solution was dispensed on the printing plate with engraved pattern of 120 lines cm^−1^, then printed at a speed of 18 m min^−1^. The freshly printed film was immediately immersed in a bath filled with diethyl ether, followed by N_2_ drying. The perovskite layers were finally formed by annealing at 100 °C for 10 min. For the demonstration of a flower‐pattern printing, 0.8 wt% of PVP (K90, Sigma‐Aldrich) was dissolved with 1.2 g of perovskite precursors in 1 mL of solvents (DMF:DMSO = 8:1). For the two‐step process, 0.6 g of PbI_2_ was dissolved in a mixed solvent of DMF and DMSO at a ratio of 9:1, then spin‐coated by 2000 rpm for 30 s, or printed with a printing plate with line density of 100 lines cm^−1^ at a speed of 18 m min^−1^. The PbI_2_ film was immersed in a bath filled with mixture of IPA and water for 10 s, then blowed by compressed air. The perovskite layer was formed by dipping of the porous PbI_2_ layer in a MAI bath (40 mg mL^−1^) for 3 min followed by drying at 100 °C for 10 min.

*Fabrication of Gravure‐Printed, Flexible PSCs at a Lab‐Scale*: A ITO‐coated PET roll was used as a substrate (Eastman Flexvue OC50). The ITO was selectively patterned by rotary screen printing of an etching paste (HiEP‐300, P&P Solution Co., Ltd.).^66^ The roll was used without further purification for the R2R process and for the laboratory‐scale printing tests, by cutting into individual sheets. The printing of all the layers at a laboratory scale was conducted by a table‐top, gravure printing machine (Labratester, Norbert Schläfli Maschinen). First, SnO_2_ nanoparticles (20% in deionized (DI) water, Alfa Aesar) was diluted by a mixed solution of water and IPA (Sigma‐Aldrich). The SnO_2_ solution was then printed by a printing plate with an engraved pattern having a line density of 120 lines cm^−1^, at a speed of 18 m min^−1^. The resulting films were annealed in a convection oven at 120 °C for 5 min. The perovskite layer was formed by either one‐ or two‐step process, as described above. A Spiro‐OMeTAD was dissolved in chlorobenzene (0.0909 g mL^−1^), with 23 µL of bis(trifluoromethane)sulfonimide lithium salt (Li‐TFSI)/acetonitrile (540 mg mL^−1^), 39 µL of 4‐*tert*‐butylpyridine (*t*BP, Sigma‐Aldrich), and 10 µL of tris(2‐(1H‐pyrazol‐1‐yl)‐4‐*tert*‐butylpyridine)cobalt(III) tri[bis(trifluoromethane)sulfonamide] (FK209, LumTec)/acetonitrile (0.376 g ml^−1^). For the spin‐coated device, the Spiro‐OMeTAD solution was spin‐casted at 2000 rpm for 30 s. For the gravure printing, the Spiro‐OMeTAD solution was printed by the printing plate of engraved pattern of 100 lines cm^−1^. A P3HT (#4002‐E, Rieke Metals) was dissolved in chlorobenzene at a concentration of 50 mg mL^−1^, then printed with a printing plate engraved with a line density of 80 lines cm^−1^. Li‐TFSi solution (540 mg mL^−1^) and *t*BP were added to the P3HT solution at an amount of 4 µL each. Ag electrodes were formed by thermal evaporation with a shadow mask to define the cell area.

*R2R Printing of PSCs*: The R2R printing of perovskite solar cells was conducted on the patterned ITO‐coated PET roll, by using a custom‐built pilot‐scale R2R printing machine. The patterned ITO was plasma‐treated (Ar/N_2_), then the SnO_2_ nanoparticles were gravure printed at a speed of 8 m min^−1^ followed by annealing with hot air drying unit at 120 °C for 30 s. The PbI_2_‐DMSO solution was gravure printed with a speed of 4 m min^−1^ on top of the plasma‐treated (Ar/N_2_) SnO_2_ layer. The substrate was then guided through a bath filled with water and IPA (1:1 by volume) for 30 s. The resulting PbI_2_ roll was moved to laboratory, cut into individual sheets, and dipped in a MAI bath for 3 min. The converted perovskite layers were annealed at 100 °C for 10 min. The printing of Spiro‐OMeTAD or P3HT layers, and the thermal evaporation of the Ag electrode was processed identically as for the laboratory printed devices.

*Characterization*: The UV–vis absorption spectra were obtained by UV–vis spectrophotometer (Shimadzu UV‐2550). The top‐view images of all the layers constituting perovskite solar cells and cross‐sectional image of flexible perovskite solar cell were obtained by using field emission SEM (Mira 3 LMU, Tescan) operated at 20 kV. X‐ray diffraction spectra were obtained by using Rigaku smartlab X‐ray diffractometer. The *J*–*V* curves and MPPT tracking curve were obtained by using a solar simulator (Newport, Oriel Class A, 91195A) with voltage sourcemeter (Keithley 2420) under 100 mA cm^−2^ illumination with standard AM1.5G condition. Light intensity was calibrated by Si reference cell certified by NREL, USA. The samples were all masked with metal mask having an active area of 0.052 cm^2^. EQE was measured using a power source (Newport 300 W Xenon lamp, 66920) with a monochromator (Newport Cornerstone 260) and a multimeter (Keithley 2001). AFM images were obtained by Veeco Nanoscope IV Multimode AFM (Digital Instruments).

## Conflict of Interest

The authors declare no conflict of interest.

## Supporting information

Supplementary
