# Supplementary material for: Gravure‐Printed Flexible Perovskite Solar Cells: Toward Roll‐to‐Roll Manufacturing
Source: Adv Sci (Weinh). 2019 Jan 28;6(7):1802094. doi: 10.1002/advs.201802094 (PMC6446604; doi:10.1002/advs.201802094)
Supplement: Supplementary file 1 — Supplementary [file ADVS-6-1802094-s001.pdf]

Copyright WILEY-VCH Verlag GmbH & Co. KGaA, 69469 Weinheim, Germany, 2019.

ADVANCED SCIENCE Open Access

Supporting Information

for *Adv. Sci.,* DOI: 10.1002/advs.201802094

Gravure-Printed Flexible Perovskite Solar Cells: Toward Roll-to-Roll Manufacturing

*Young Yun Kim, Tae-Youl Yang, Riikka Suhonen, Marja Välimäki, Tiina Maaninen, Antti Kemppainen, Nam Joong Jeon, and Jangwon Seo**

Copyright WILEY-VCH Verlag GmbH & Co. KGaA, 69469 Weinheim, Germany, 2016.

Supporting Information

Gravure printed flexible perovskite solar cells: towards roll-to-roll manufacturing

Young Yun Kim†, Tae-Youl Yang†, Riikka Suhonen, Marja Välimäki, Tiina Maaninen, Antti Kemppainen, Nam Joong Jeon, Jangwon Seo*

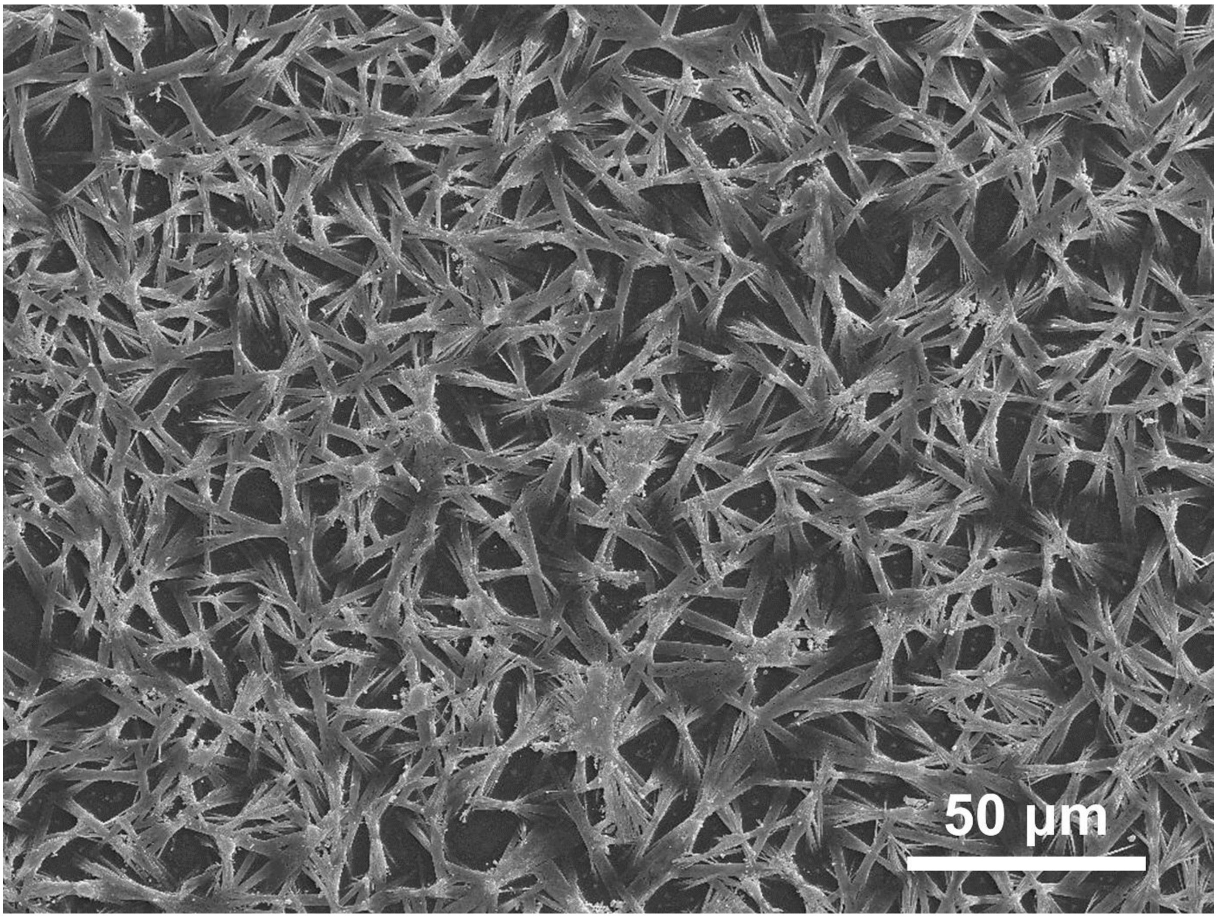

Figure S1. SEM image of perovskite formed after 60 s of drying.

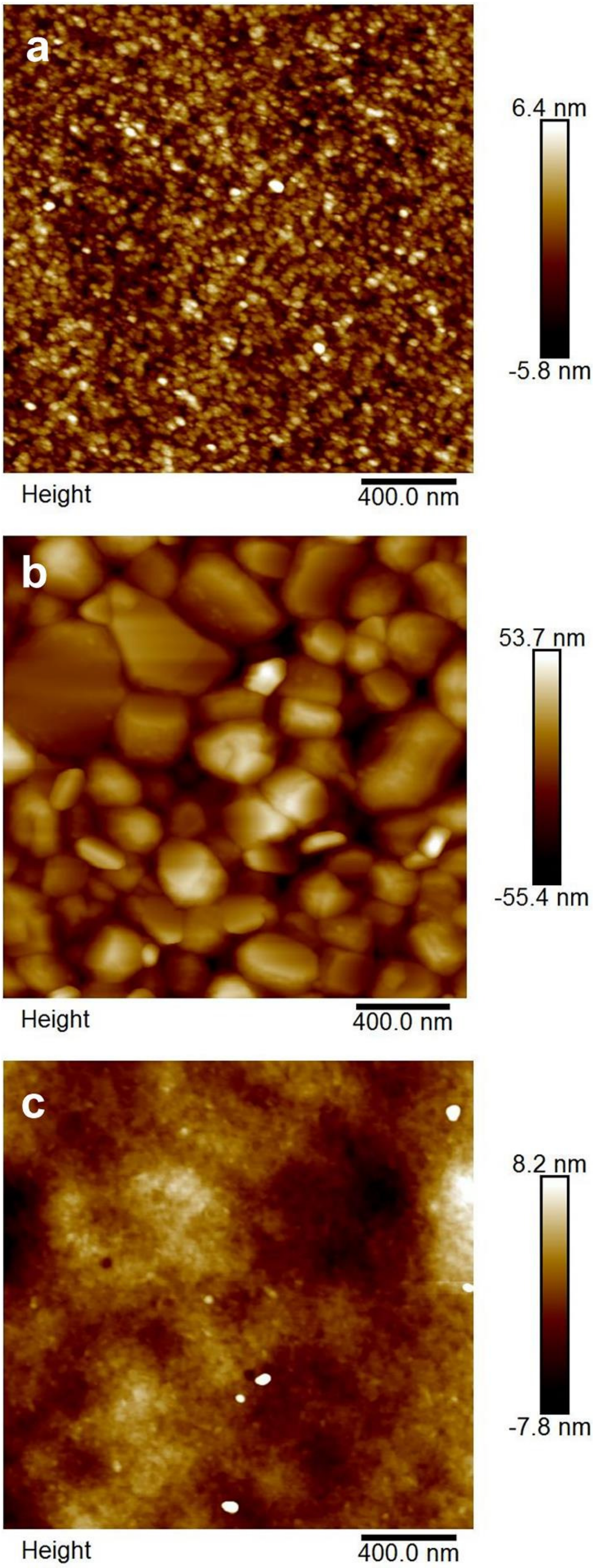

Figure S2. AFM images of printed a) SnO_2, b) perovskite, and c) Spiro-OMeTAD.

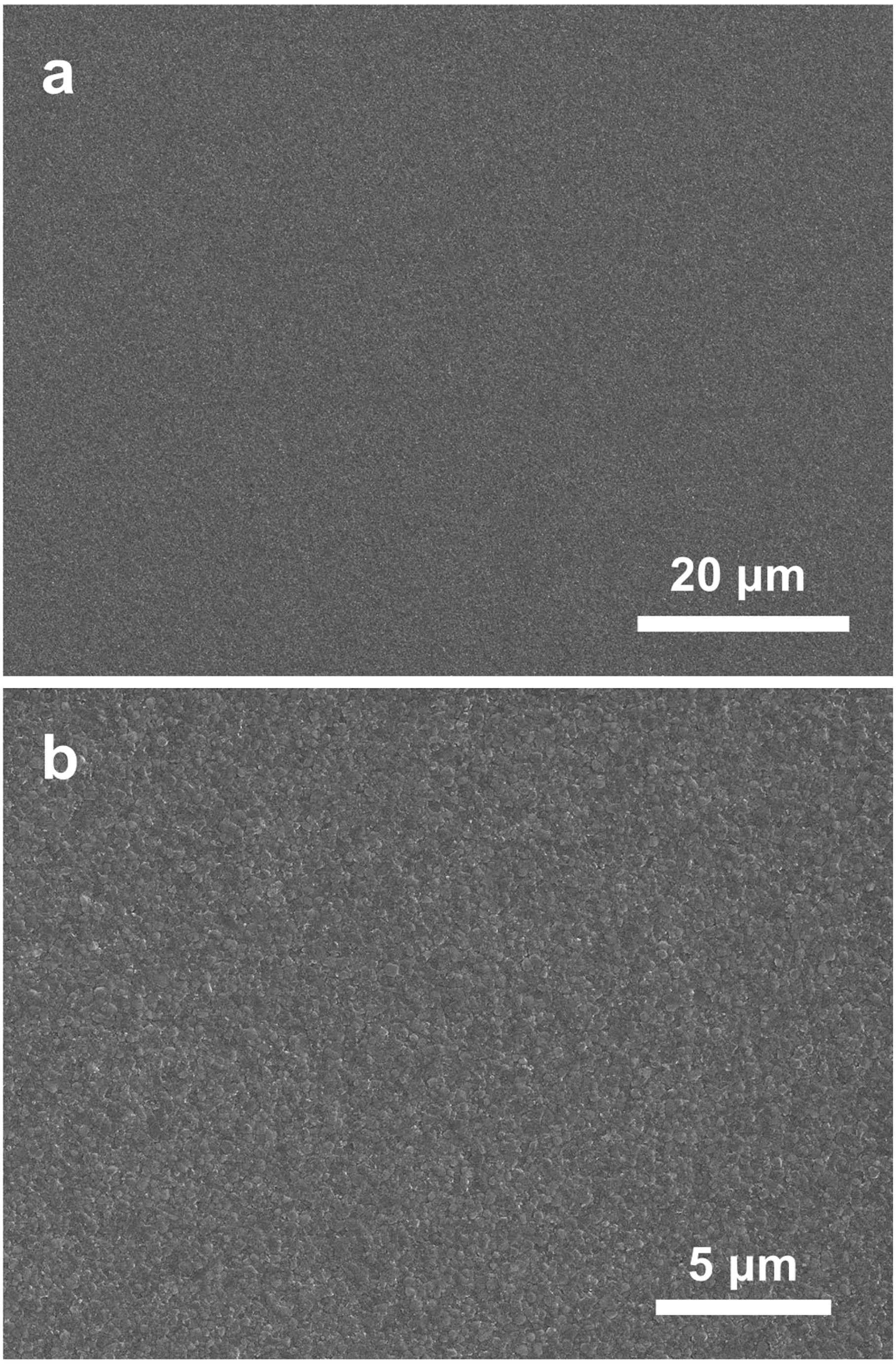

Figure S3. SEM images of gravure printed perovskite layer at a magnification of a) 3k, b) 10k.

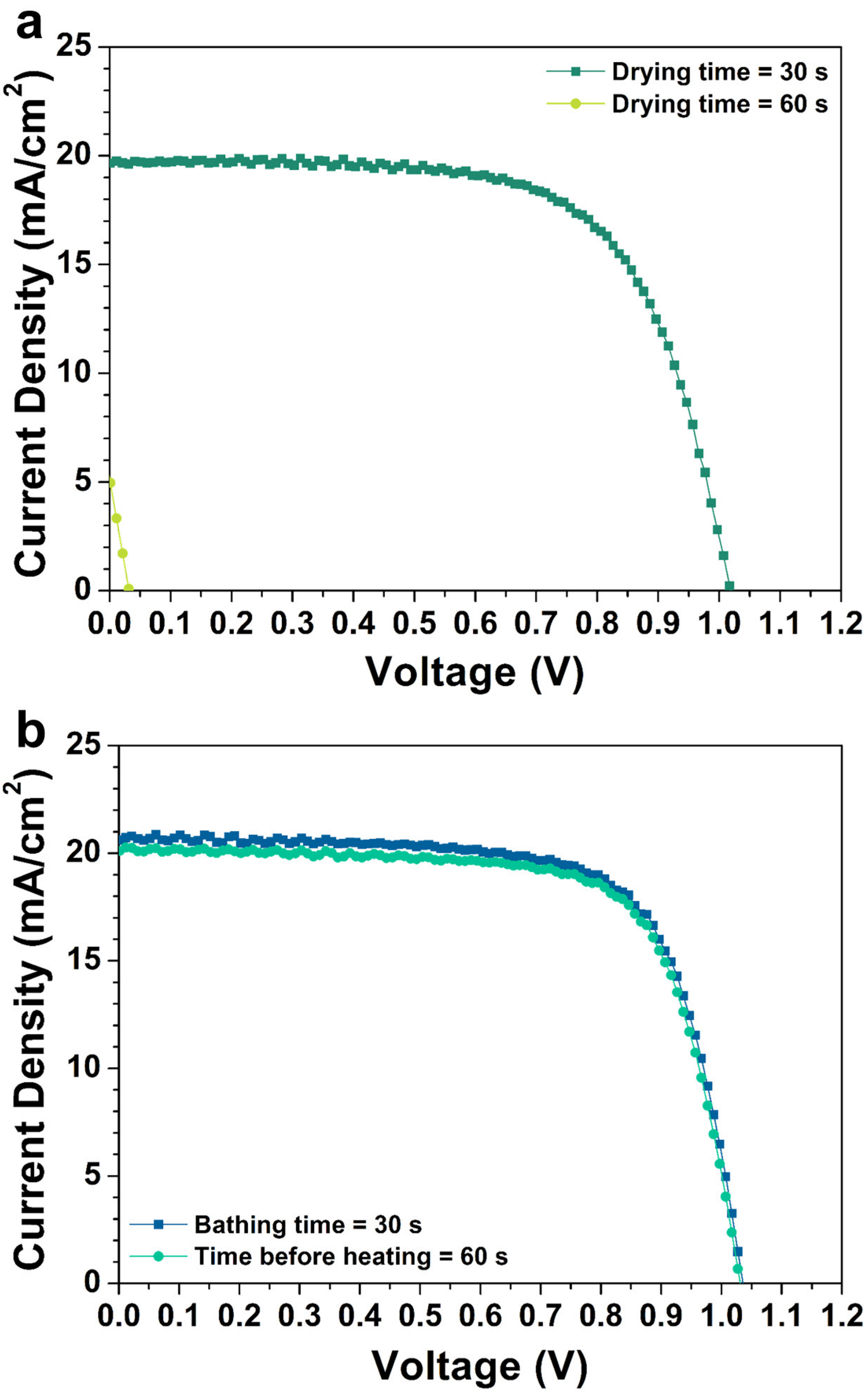

Figure S4. J-V curves of flexible printed PSCs made by varying a) drying time and b) bathing time and time between bathing and heating.

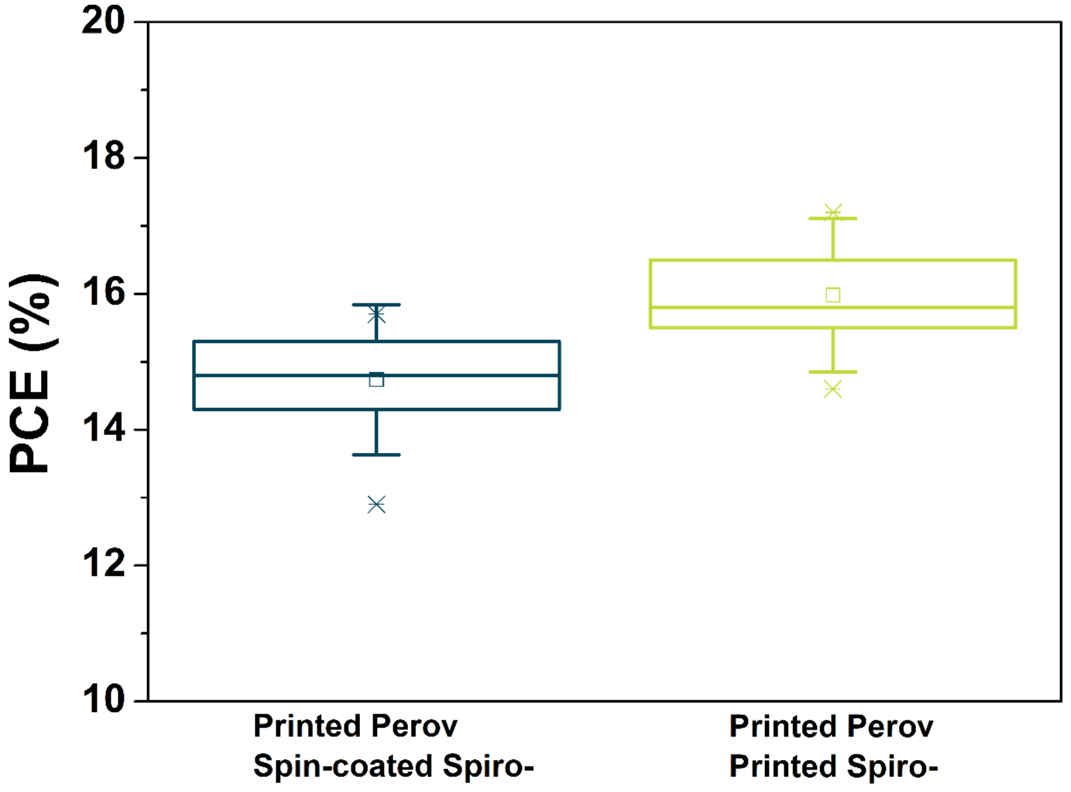

Figure S5. Box chart of PCE for devices with printed and spin-coated Spiro-OMeTAD.

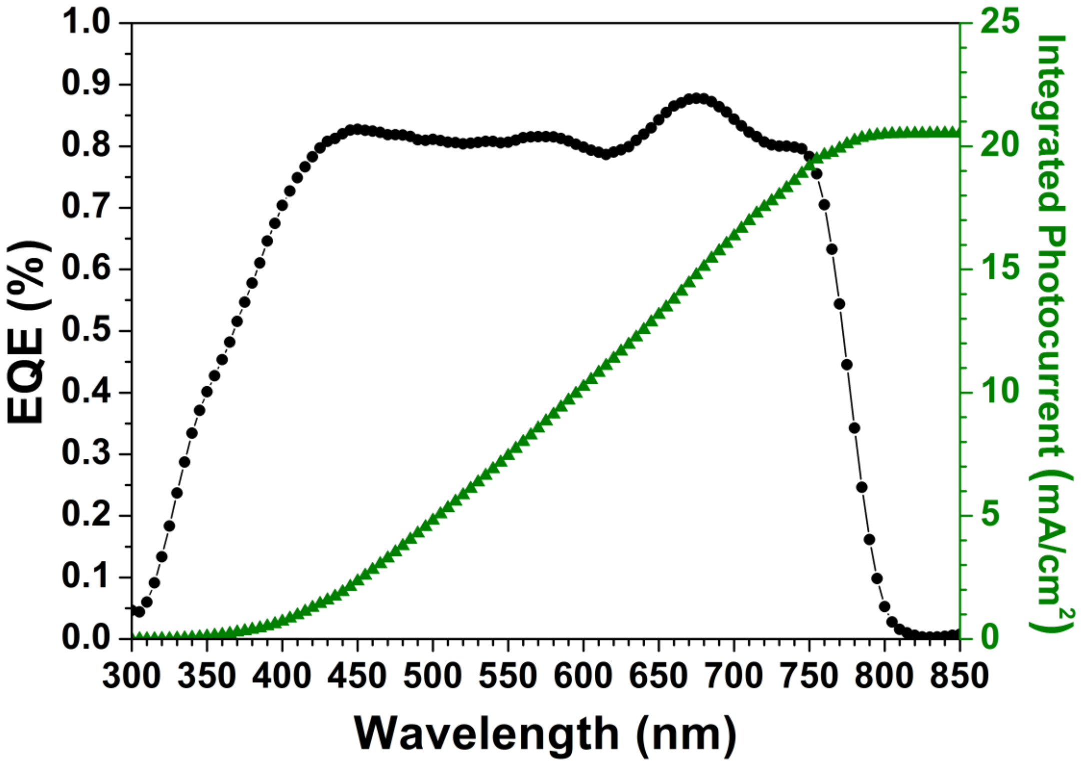

Figure S6. EQE spectra of all-printed flexible PSC.

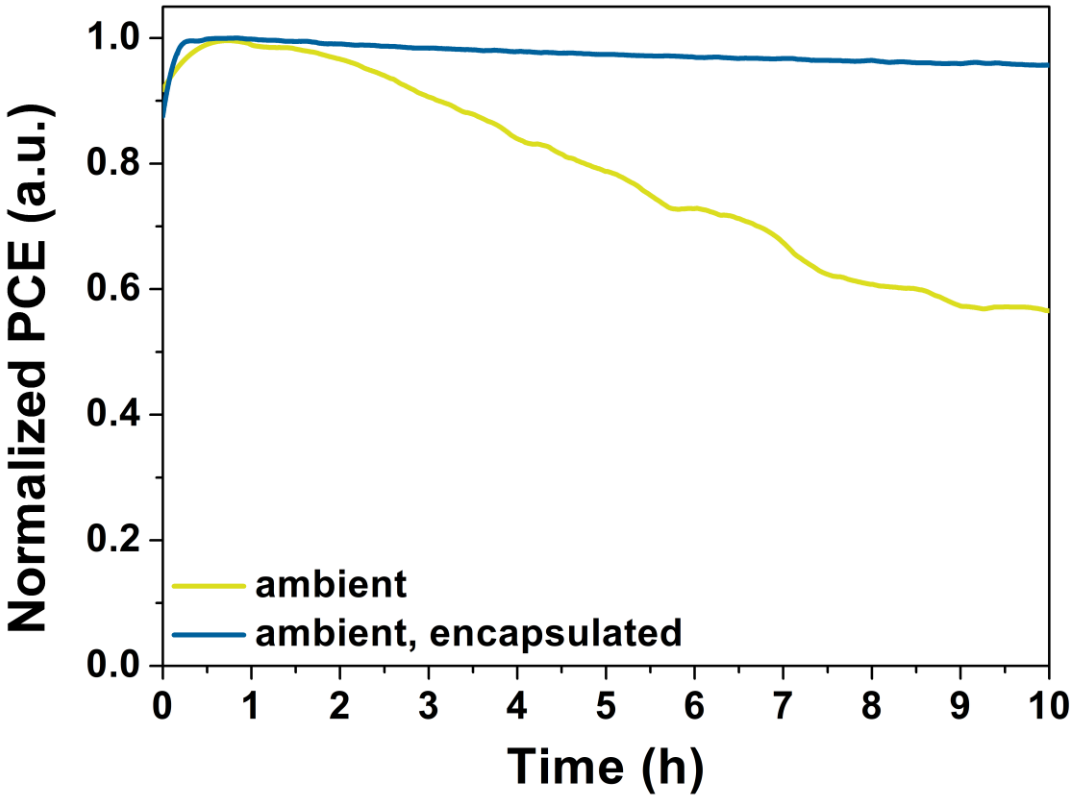

Figure S7. The evolution of MPPT efficiency under constant illumination for the gravure printed devices with and without encapsulation.

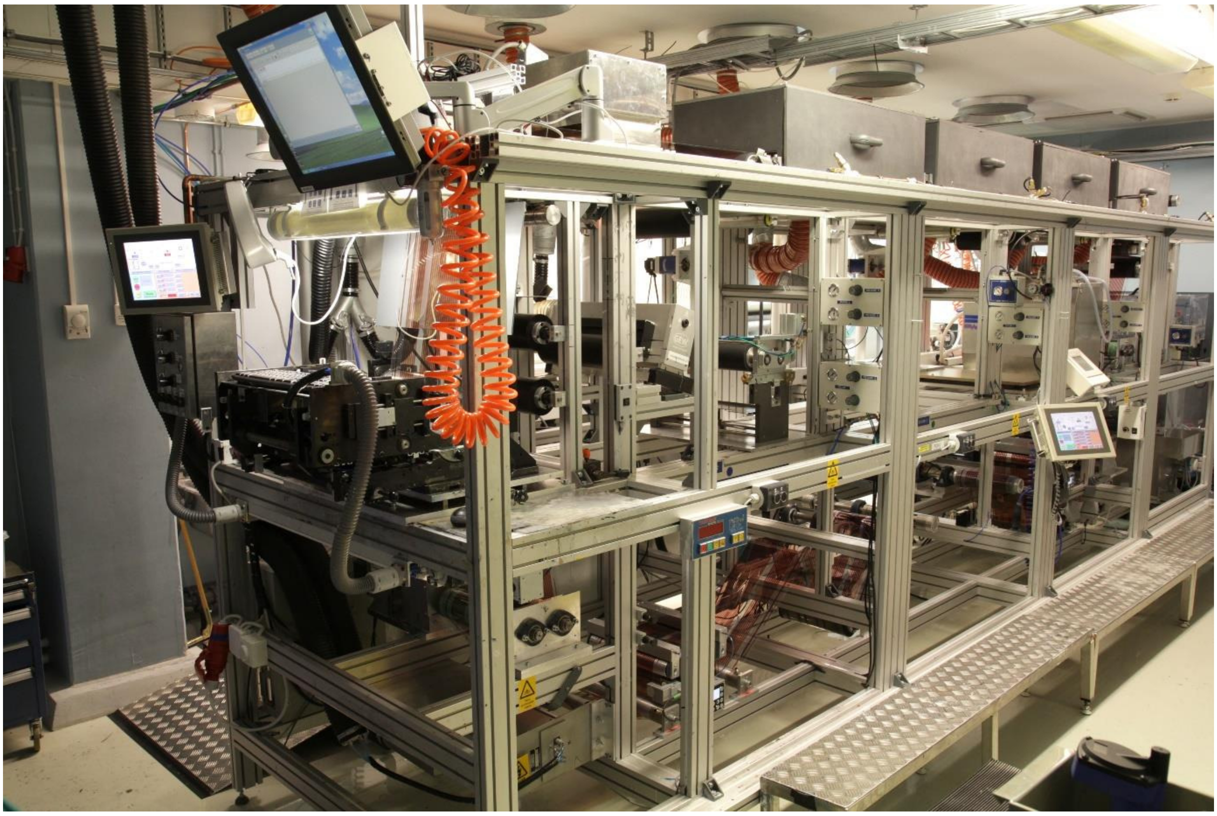

Figure S8. Picture of pilot R2R printing machine.

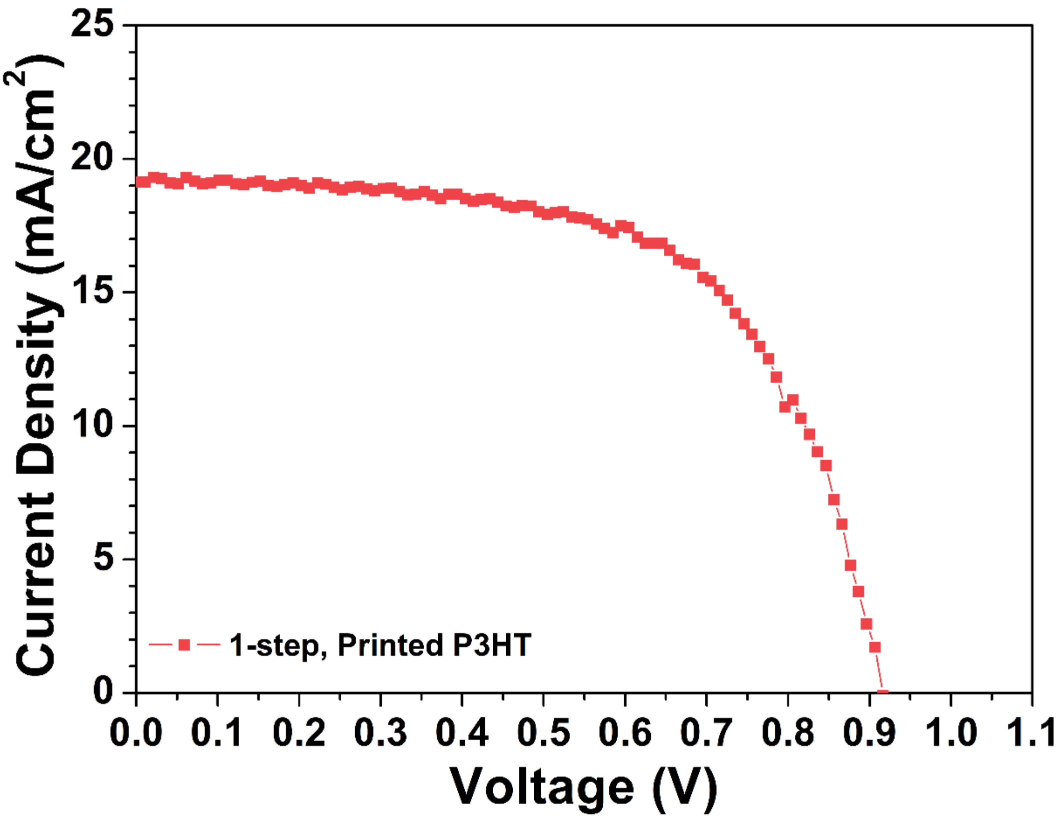

Figure S9. J-V curve of flexible perovskite solar cell with one-step process, printed P3HT HTL.

Table S1. The solar parameters of the flexible PSCs with different timescales.

Samples [a)]	V_{oc} [V]	J_{sc} [mA/cm^2]	Fill factor [%]	Efficiency [%]
Drying time = 30 s	1.02	19.7	67.1	13.4
Drying time = 60 s	0.03	5.0	24.1	0.04
Bathing time = 30 s	1.03	20.6	71.4	15.3
Time before heating = 60 s	1.03	20.1	72.3	14.9

[a)] PET/ITO/Printed SnO_2/Spin-coated perovskite/Spin-coated Spiro-OMeTAD/Ag

Table S2. The solar parameters of the two-step processed PSCs with different ratios of solvents.

Samples [a)]	V_{oc} [V]	J_{sc} [mA/cm^2]	Fill factor [%]	Efficiency [%]
IPA:water = 10:0	1.09	22.8	72.4	18.0
IPA:water = 7:3	1.06	23.6	70.8	17.9
IPA:water = 5:5	1.06	22.7	71.2	17.3
IPA:water = 3:7	1.06	22.9	67.4	16.5
IPA:water = 0:10	0.78	14.8	46.0	5.3

[a)] Glass/ITO/SnO_2/$MAPbI_3$/Spiro-OMeTAD/Au
